# Effects of growth stage and seasons on the phytochemical content and antioxidant activities of crude extracts of *Celosia argentea L.*

**DOI:** 10.1016/j.heliyon.2020.e04086

**Published:** 2020-06-02

**Authors:** O.D. Adegbaju, G.A. Otunola, A.J. Afolayan

**Affiliations:** Medicinal Plants and Economic Development (MPED) Research Centre, Department of Botany, University of Fort Hare, Alice 5700, South Africa

**Keywords:** Food science, Nutrition, *Celosia argentea*, Antioxidants, Oxidative stress, Phytochemical, Growth stages

## Abstract

This study investigated the phytochemical constituents and antioxidant properties of crude extracts of *C. argentea* at different maturity stages and seasons. Total phenols, flavonoids, and proanthocyanidin content from water, acetone and methanol extracts were evaluated spectrophotometrically. The antioxidant activities were measured using 2,2- diphenyl-1-picrylhydrazyl (DPPH), 2,2′-azino-bis (3-ethylbenzthiazoline-6-sulfonic acid) (ABTS), Ferric reducing antioxidant power (FRAP) and Total antioxidant capacity (TAC) models. Results showed that the flowering stages in all the solvent extracts gave the highest polyphenolic content with the acetone extract significantly higher than the methanol and aqueous extracts (P < 0.05). The highest value for total polyhenolic content 80.75 ± 4.21 for the first trial and 89.69 ± 2.13 μg/mL in the second trial; while the flavonoids was 874.76 ± 7.87 and 946.19 ± 7.87 μg/mL in the first and second trials respectively; and proanthocyanidin content was 170.00 ± 0 and 100.90 ± 1.29 μg/mL. Overall, the aqueous extracts had the lowest content of all the phytochemicals. The antioxidant activities ranged from low to high at different growth stages of the plant. While low to no activity was observed in the aqueous extracts in all the assays, the methanol extracts of the flowering stages showeds the best activity in the first and second trials with IC_50_ values of 104.10 ± 8.59 and 120.02 ± 13.37 μg/mL respectively in ABTS. Similar trend was obtained in the DPPH assay with the highest activity in the methanol flowering extract with IC_50_ of 52.36 ± 0.76 μg/mL (first trial) and 49.36 ± 0.29 μg/mL (second trial). The FRAP and TAC also had the highest activity in the flowering stages in all solvents, but with the acetone extracts having the overall inhibition on both radicals. This study revealed that *Celosia argentea* phytoconsituents and antioxidant potential can be influenced by physiological and developmental stages of the plant.

## 1 Introduction

Increased exposure to the environment, higher levels of dietary xenobiotics and cellular metabolism leads to the production of free radicals in the body. Free radicals form reactive oxygen species (ROS) such as the hypohalite, super oxide anion, hydroxyl radicals and hydrogen peroxide. These radicals have been directly connected to various pathological conditions including the increase in pathogenesis of atherosclerosis, diabetes mellitus, multiple sclerosis, aging and several other oxidative damages (Nimse and Pal, 2015). However, the negative cellular effect of these radicals can be countered by the actions of antioxidants. Antioxidants are molecules that can considerably impede or foil the toxic oxidative processes, even at very low concentrations. They can either be synthesized *in vivo* (reduced glutathione, superoxide dismutase and catalase) or consumed as dietary supplements such as vitamin C, selenium, vitamin E and carotenoids (Kasote et al., 2015). Supplementation with dietary antioxidant (either as food or herb) has gained greater recognition because of their ability to hinder the progressive production of free radicals, obstruct the actions of ROS-generating enzymes (nitric oxide, synthase and xanthine oxidase) and improve the endogenous cellular antioxidant mechanisms such as the up-regulation of the activity of catalase and super oxide dismutase (Kurutas, 2015).

Medicinal and food plants are natural reserves of several biologically active compounds. These phytoconstituents have been reported to exhibit various biological effects such as anti-inflammatory, anti-carcinogenic, hypoglycemic, anti-urolithiatic activities etc (Unuofin et al., 2018).

One of the various food and medicinal plant genera that have been exploited for various nutritional, folkloric medicines and pharmacological application is *Celosia.* These plant species are mostly consumed as vegetables and as food supplements in combating micronutrients deficiencies (Gupta et al., 2013). Notable among the species is *Celosia argentea L.* This plant is traditionally used as folk medicines in most part of the world for the management of several disorders such as, jaundice, diarrhea, mouth sores, fever and inflammation (Nidavani et al., 2014). Other pharmacological functions includes anti-diabetes, anti-hypertensive hepatoprotective, anti- inflammation, anti-bacterial and immunostimulatory (Tang et al., 2016; Varadharaj and Muniyappan, 2017). However the specific stage of maturity at which these bioactive contents and biological activities are most prominent is yet to be elucidated.

Biosynthesis of phytochemicals in plant is majorly dependent on their genetic make-up. However, the concentration of many oxidative phytochemicals is significantly affected by farming practices and other environmental stress conditions such as seasonal changes, geographical location, plant maturity, soil type and post harvet processing to mention just a few. Therefore, breeding of plants with high concentration of phytochemical constituents and antioxidant capacity depends solely on these factors (Tsao et al., 2006).

In this context, the aim of this study is to evaluate the effect of season (summer and winter), growth stage and solvent on the polyhenolic content and the corresponding antioxidant activities of *C. argentea L.,* in order to ascertain the best growth stage at which this species possesses the highest phytochemical components and most potent antioxidant capacity.

## 2 Materials and methods

### 2.1 Plant material and location

Mature seeds of *Celosia argentea* L. were obtained from an Agro shop in Nigeria. Seeds were planted in plastic pots filled with compost soil (Kanya Nursery, Alice Eastern Cape, South Africa) in the glass house of the University of Fort Hare, Alice, 5700 Eastern Cape, South Africa. The university geographical location lies at latitude 32^o^ 47′- 19^o^26′ S; longitude 26^o^50′- 42^o^306′E and at altitude of 514.70 m above sea level. Three harvest periods: pre-flowering PRE, flowering FLW and postflowering PST stages was employed. Fresh leaves and stem were harvested when the first flower appeared (preflowering stage (PRE), 7–10 g per pot), the second harvest was done when 50 percent of all the plants had flowered (flowering stage (FLW), 20–25 g per pot) while the third harvest was done when the flowers were few and dropping (post-flowering period (PST), 20–30 g per pot). For each harvest period, a total of 27 pots were harvested.

The seeds were planted in during summer (October 2017 to January 2018) and winter (March to May 2018) and the herbarium sample was authenticated by Prof. C. Cupido while a sample voucher (Ade/med/2017/01) was submitted to the Giffen Herbarium, University of Fort Hare.

### 2.2 Preparation of extracts

After each harvest, the aerial part of the plant (stem and leaves for preflowering and stem, leaves and flowers for flowering and postflowering) were rinsed with distilled water and blotted dry with a paper towel. Thereafter, they were oven-dried at 40 °C for 72 h until fixed weight was achieved. The dried leaves were pulverized with an industrial electric blender (Hamilton Beach, HBF500s series, Canada). To prepare crude extract of the sample, 200 g of the ground sample were agigtated constantly for 72 h using a shaker (Gallenkamp incubator orbital shaker) in 1.5 L of distilled water, acetone and methanol. The solution was thereafter filtered using Whatman No. 1 filter paper, a Buchner funnel and a vacumm pump. The aqueous extract (APRE, AFLW and APST) was freezed at −40 °C with a shell freezer and freeze-dried (Vir Tis Co, Vir Tis benchtop K, Gardiner, NY). The solvents acetone (AcPRE, AcFLW an AcPST) and methanol (MPRE, MFLW and MPST) were vaporized using a rotary evaporator (Strike-202 Steroglass, Italy), at their respective boiling points. The extaction solvents were chosen based on their extractive abilities and solubility. Water was chosen as one of the solvents of extraction because it is the medium used for cooking and preparing decoctions of medicinal plants in folklore medicine. Methanol was used because of its ability to extract polar and non-polar substances. Also, because of the lower boiling point of methanol (64.7 °C) when compared to ethanol (78.4 °C), lower temperature is required to evaporate the solvent from the methanol extract in the rotary evaporator; hence the extract is less damaged. Acetone was chosen because of it polar and non-polar characteristics. The crude extracts were stored at 4 °C in a refrigerator (Polyscience AD15R-40-A12E, USA) until needed. Percentage yield of solvent was determined and recorded. All dry extract samples were reconstituted in methanol (1mg per ml) to prepare the stock solution needed for various antioxidant assays. The mixture was then sonicated (Branson 2510, 220–230V, 50–60Hz, 130W and 0.6A; Branson Ultrasonics Corporation, DANBURY, CT06813, USA) for 40 min at 27 °C. The mixture was vortexed thoroughly and dissolved samples were used for all analyses in the study.

### 2.3 Solvents and reagents

Chemicals and solvents used including ascorbic acid, sodium nitrite, aluminum trichloride, n-butanol, diethyl ether, sodium carbonate, FolinCiocalteu solution, 2,2′-azino-bis(3-ethylbenzthiazoline-6-sulphonic acid, ammonium molybdate, sodium hydroxide, sodium chloride 2,2- diphenyl-1-picrylhydrazyl, AlCl_3_, potassium acetate, BHT, vanillin, ammonia solution, ferric chloride, acetone, gallic acid, catechin, quercetin, methanol, sodium nitroprusside, potassium ferricyanide, sodium phosphate, rutin, trichloroacetic acid, acetic acid (glacial), phosphate buffer and hydrochloric acid. All the chemicals used in this study were of analytic grade and were purchased from Merck and Sigma-Aldrich, Gauteng, South Africa.

### 2.4 Quantitative phytochemical evaluation

#### 2.4.1 Total phenolic content

The total phenolic content was determined using Folin-Ciocalteu's reagent by method described by Agbor et al. (2010) with slight modifications to the concentration. A 0.5 mL aliquot of the different extracts or standard (gallic acid, 1000 μg/mL) was mixed with 2.5 mL of 10% (v/v) Folin-Ciocalteu's reagent and allowed to incubate for 5 min, after which 2 mL of 7.5% (w/v) anhydrous Na_2_CO_3_ was then added to the solution. The mixture was incubated for 30 min at 40 °C. The absorbance was measured at 765 nm using Hewlet Packard VR-2000 spectrophotometer. A standard curve was prepared using a concentration range of 20–100 μg/mL of gallic acid in methanol. The total phenolic content in mg gallic acid equivalent (GAE)/mL was derived from the standard curve equation; y = 0.0088x + 0.0591, R^2^ = 0.9993, and expressed in μg GAE/mg using the formular y = C V/m; where

C = concentration obtained from standard curve in μg/mL
V = volume of plant extract used in the assay in mL
m = mass of extract used in the assay in mg.

#### 2.4.2 Flavoniods

The total flavonoid content was determined using the colometric aluminum chloride method proposed by Lamien-Meda et al. (2010). The production of yellow-orange coloration as a result of the interaction of aluminum chloride with flavonoids was the basis of quantification for this method. Briefly 0.5 mL of the extracts, quercetin and the control at four concentrations (200, 400. 800 and 1000 μg/mL), were placed in different test tubes. 2 mL of deionized water and 0.15 mL of sodium nitrite (5% w/v) were then added to the mixture. The solution was allow to stand for 6 min, after which 0.15 mL of 10% aluminium chloride was added to the mixture and incubated for 5 min at 25 °C. 1 mL of 1 M sodium hydroxide was added to the solution and made up to 5 mL by adding 1.2 mL distilled water. Absorbance was measured using a spectrometer at 420 nm. The control solution was used as blank; and the experiment was done in three replications. The flavonoid content was calculated using the calibration curve equation; y = 0.0014x, R^2^ = 0.9994 and expressed as μg of quercetin equivalent (QE)/mg as described earlier for the phenolics.

#### 2.4.3 Proanthocyanidin

Total proanthocyanidin content was assessed based on the procedure described by Ohikhena et al. (2018) with slight modification. To 0.5 mL of the extract (1000 μg/mL) Concentration range of 200 μg/mL to 1000 μg/mL of catechin (standard drug) and solvent of dissolution (control), was added to 3 mL of vanillin - methanol (4% w/v) and 1.5 mL of hydrochloric acid and vortexed. The mixture was allowed to stand for 15 min at room temperature. The control solution was used as blank. The absorbance was measured at 500 nm using a UV- 3000 PC spectrophotometer. Proanthocyanidin content was evaluated using the calibration curve equation: y = 0.0011x, R^2^ 0.9943 and was expressed as μg catechin equivalent (CE)/mg using the formula, CV/m as earlier mentioned in phenol.

### 2.5 *In- vitro* anti-oxidant assays

The antioxidant capacity of leaves and stem of *C. argentea* were determined using DPPH, ABTS, ferric reducing antioxidant power (FRAP) and phosphomolybdate (Total antioxidant capacity).

#### 2.5.1 (2,2-diphenyl-1-picrylhydrazyl) DPPH radical scavenging activity assay

DPPH free radical scavenging assay was determined as outlined by Unuofin et al. (2018). 1 mL of 0.135 mM DPPH radical was mixed with 1 mL each of the test samples (previously prepared with methanol), standard antioxidant (BHT) and control at four concentrations (62.5, 125, 250 and 500 μg/mL). Subsequently, the mixture was vortexed and kept in the dark 25 °C for 30 min. The rate of absorbance was then spectrophotometrically measured at 517 nm, using methanol as the blank and positive control. Finally, the scavenging ability of the plant extract was calculated using the equation:

DPPH scavenging activity (%) = [(Abs control – Abs sample)/ (Abs control)] ×100;

Where; Abs control is the absorbance of DPPH + methanol; Abs sample is the absorbance of DPPH radical + sample/or standard.

#### 2.5.2 (2, 2′-azino-bis (3-ethylbenzothiazoline)-6-sulfonic acid) ABTS radical scavenging activity

The method described by Khan et al. (2012) was used to determine the ABTS radical scavenging activity of the plant extract with little modifications. ABTS stock solution was prepared by mixing 7 mM ABTS and 2.45 mM potassium persulfate in equal quantity. The resultant stock was incubated in the dark for 18 h at 25 °C to form a green-coloured ABTS radical (ABTS^+^). This solution was further diluted by mixing 1 mL of the ABTS^+^ solution with 50 mL of methanol to obtain a working solution with absorbance of 0.700 ± 0.006 at 734 nm. The extracts and standards (Rutin and BHT) of concentrations ranging from 12.5 to 200 μg/mL were then mixed with 1 mL of ABTS solution and kept in the dark for 7 min. Absorbance was measured at 734 nm and the percentage inhibition of ABTS^+^ by the extract was calculated from the equation:

% inhibition = [(Abs control – Abs sample)] / (Abs control)] × 100.

#### 2.5.3 Ferric reducing power (FRAP)

The ferric reducing power of the plant extracts was determined by using the method described by Jayanthi and Lalitha (2011). The FRAP mixture consisted of 2.5 mL of 0.2 M phosphate buffer reagent (Mixture of 62.5% monobasic and 37.5% dibasic: pH 6.6) and 2.5 mL of 1% potassium hexa-cyanoferrate (w/v). To this was added 1mL of the standard (Gallic acid and ascorbic acid) and the *C. argentea* extracts at five concentrations (50, 100, 200, 500 and 1000 μg/mL). The mixture was incubated at 50 °C for 20 min in a water bath. Subsequently, 2.5 mL of 10% Trichloroacetic acid (TCA) (w/v) was added to the mixture, after which the mixture was centrifuged for 10 min at 3000 rpm. Then, 2.5 mL of distilled water and 0.5mL 0.1% freshly prepared FeCl_3_ were added to the supernatant from the centrifuged solution. The resultant solution was allowed to stand for 10 min at 25 °C. The percentage inhibition of the radicals by the plant sample was measured at 700 nm against the methanol blank.

Ferrous sulphate equivalent concentration in mM was calculated from the standard graph and expressed as mg Fe (II) equivalent/mg.

% inhibition = [(absorbance of sample – absorbance of control)/ (absorbance of sample)] × 100

#### 2.5.4 Phosphomolybdenum assay (total antioxidant capacity,TAC)

The complex formation method using phosphomolybdenum as described by Olugbami et al. (2015) was used for the determination of the total antioxidant capacity. Briefly, 0.3 mL of the plant extract and standards (Gallic acid and ascorbic acid) at five concentrations (50, 100, 200, 500 and 1000 μg/mL) was added to 3 mL of reagent solution (4 mM ammonium molybdate, 28 mM sodium phosphate and 0.6 M sulfuric acid). The mixture was incubated at 95 °C for 90 min in a water bath. After cooling, absorbance of the solution was measured at 695 nm against the control (methanol) and the percentage inhibition was calculated as:

[(absorbance of sample – absorbance of control)/ (absorbance of sample)] × 100

#### 2.5.5 Half minimal inhibitory concentration (IC_50_)

The concentration at which 50% of the radicals were scavenged by the extracts (IC_50_) was calculated from the plot of the percentage inhibition against the concentration used. The results are expressed as half minimal inhibitory concentration (IC_50_). This is the amount of the extract or standard necessary to decrease the initial concentrations of DPPH, ABTS, FRAP radicals by 50% and the total antioxidant capacity (TAC). The lower the IC_50_ value the higher the antioxidant activity. The synthetic antioxidants (Rutin, BHT and ascorbic acid) were used as positive controls in all the assays for comparison IC50 activities between the activities of test extract and standard antioxidants.

### 2.6 Statistical analysis

All experiments were performed in triplicates. Results were expressed as means ± standard deviation (SD). Means were accepted as significantly different when data showed (P < 0.05), using the Fischer's LSD with the aid of GENSTAT 8 statistical software package.

## 3 Results

The extraction yield of different solvents from the three growth stages of both trials are shown in Table 1. The lowest yield was obtained with the acetone extracts in all the growth stages; AcPRE (35.0 g, 3.72%), AcFLW (82 g, 2.60%) and AcPST (109.5 g, 1.66%) of the first trial. Whereas, the highest yield was obtained with the aqueous extract in all growth stages; APRE (35.6 g, 22.57%), AFLW (90 g, 30.93%), and APST (110.2 g, 35.73%), followed by methanol extracts; MPRE (34.3 g, 12.78%), MFLW (79 g, 12.45%) and MPST (100.4 g, 7.66%). Apart from the increase yield observed for the acetone extract of the preflowering stage of the second trial, the same trend was observed in the resultant yield of the solvent extracts of other growth stages of the second trial.

**Table 1:** Percentage (%) yield of solvents.

	Solvents	Percentage yield (%)
First trial	APRE	22.57
	AFLW	30.93
	APST	35.73
	AcPRE	3.72
	AcFLW	2.60
	AcPST	1.66
	MPRE	12.78
	MFLW	12.45
	MPST	7.66
Second trial	APRE	17.99
	AFLW	19.45
	APST	23.46
	AcPRE	19.45
	AcFLW	2.2
	AcPST	1.78
	MPRE	13.8
	MFLW	14.5
	MPST	9.70

APRE- aqueous preflowering, AFLW- aqueous flowering, APST-aqueous postflowering; AcPRE-acetone preflowering, AcFLW- acetone flowering, AcPST- acetone postflowering; MPRE- methanol preflowering, MFLW- methanol flowering and MPST-methanol post flowering.

### 3.1 Phenolic content

**Figure 1:**
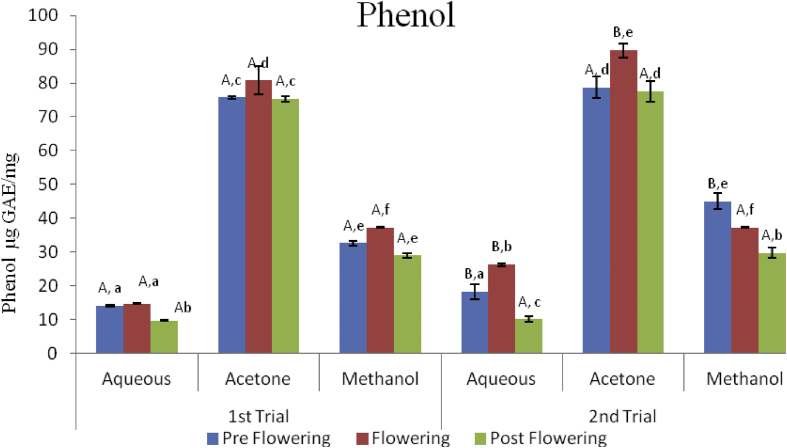
Total phenolic content of *C. argentea* extracts at different growth stages of two trials. Values are means ± SD, n = 3; P < 0.05. Different small letters within trials show significant differences P < 0.05; Different capital letters (A&B) which compare bars of a particular solvent in a particular growth phase in both trials are significantly different.

The total phenolic content (μg GAE/mg) of the different growth phases in the two trials extracted with different solvents is presented in Figure 1. The phenol content ranged from 9.76 ± 0.23–75.71 ± 0.46 μg GAE/mg in the first trial, and from 10.14 ± 0.86–78.74 ± 3.14 μg GAE/mg in the second trial. There were significant differences in the phenol content of the solvent extracts across board (both trials and growth stages). In the first trial, although the flowering stage had higher phenolic contents in all the solvent extracts, there was no statistical difference (P < 0.05) between the aqueous extracts of preflowering and flowering stages (APRE and AFLW). The preflowering and post flowering stages of the acetone (AcPRE and AcPST respectively) and methanol (MPRE and MPST) extracts also did not show any significant differences (P < 0.05). A similar trend was observed in the second trial with the flowering stage extracts higher than both pre and postflowering stages except in the methanol extracts where the preflowering stage (MPRE) had a significantly higher phenol content (P < 0.05). Also as observed in the first trial, the pre and post flowering acetone (AcPRE and AcPST) extracts did not show any significant difference. With regards to the two trials, no statistical significant differences were observed between the acetone preflowering (AcPRE); methanol flowering (MFLW); aqueous post flowering (APST); acetone post flowering (AcPST) and methanol post flowering (MPST).

Comparing the two trials with regards to the phenol content of the flowering stages, the second trial showed a significantly higher phenolic content across all the solvent used.

### 3.2 Flavoniods

**Figure 2:**
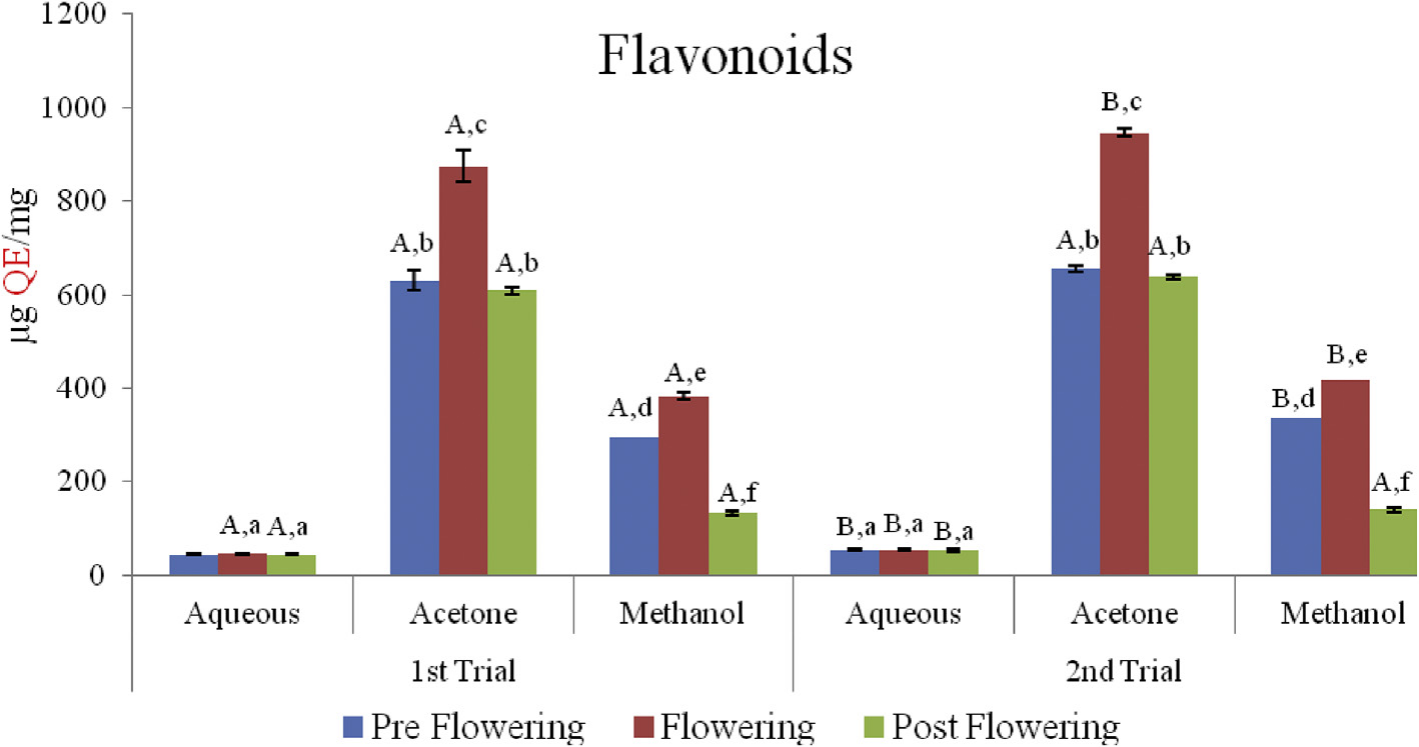
Flavonoids content of *C. argentea* extracts at different growth stages of two trials. Values are means ± SD, n = 3; (P < 0.05). Different small letters within trials show significant differences (P < 0.05); Different capital letters (A&B) which compare bars of a particular solvent in a particular growth phase in both trials are significantly different.

The flavonoid content of the extracts reported as quercetin equivalent (μg QE/mg) is shown in Figure 2. There was a consistent trend in both trials with the aqueous extracts (44.52 ± 2.18–45.48 ± 2.30 μg QE/mg in the first trial and 52.86 ± 1.89–54.05 ± 2.18 μg QE/mg in the second trial) significantly lower than the acetone and methanol extract (P < 0.05). The acetone extracts had the highest flavonoid content which ranged from 609.29 ± 34.49 to 874.76 ± 7.87 μg QE/mg in the first trial and from 637.86 ± 6.81 to 946.19 ± 7.87 μg QE/mg in the second trial.

No significant difference was observed in all three growth phases for the aqueous extracts of both trials (P < 0.05). However there were significant differences in all growth phases in both trials in the flavonoid content; with the acetone extracts of the flowering stages being the highest (P < 0.05).

### 3.3 Proanthocyanidin

**Figure 3:**
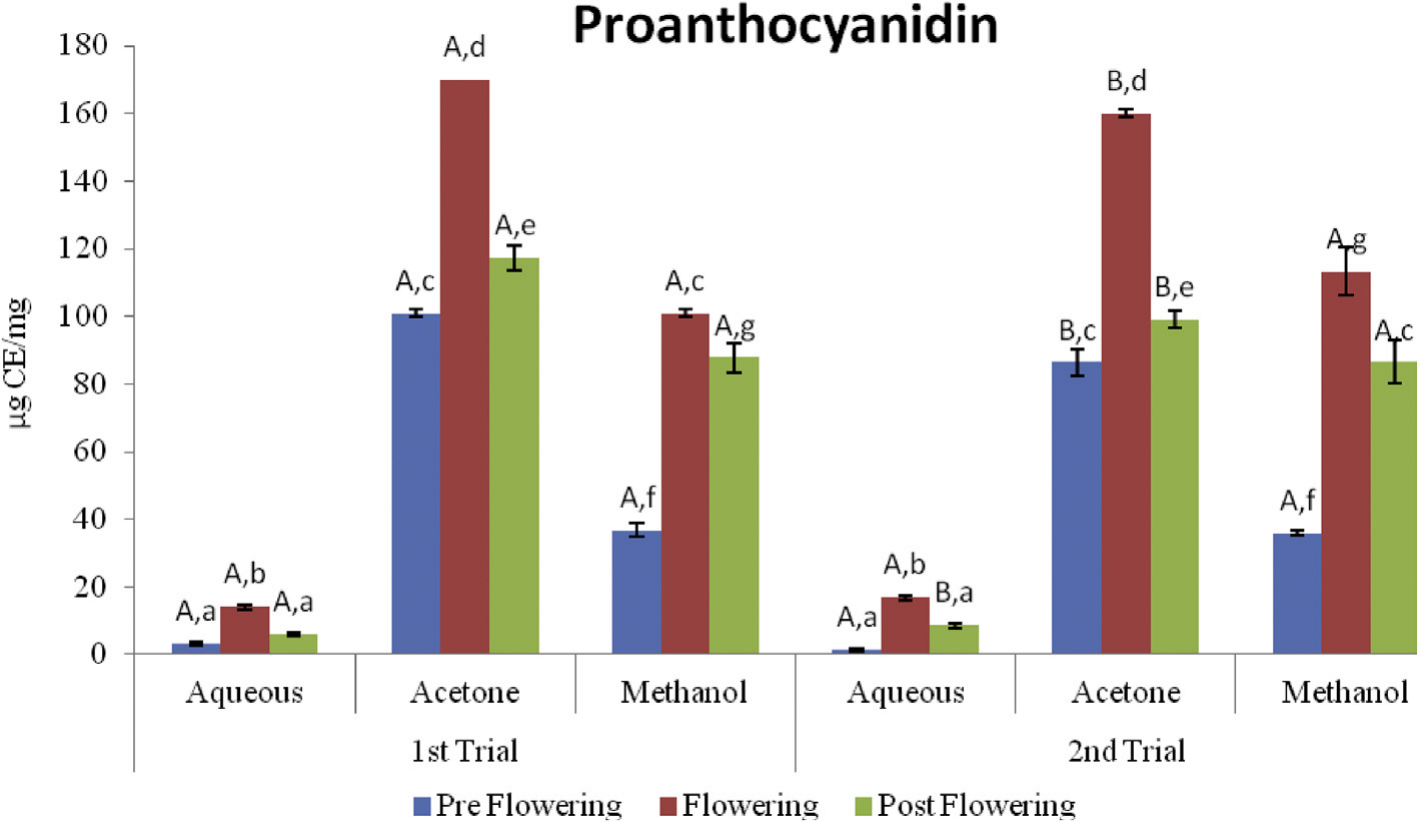
Proanthocyanidin contents of *C. argentea* extracts at different growth stages of two trials. Values are means ± SD, n = 3. Different small letters within trials show significant differences (P < 0.05) Different capital letters (A&B) which compare bars of a particular solvent in a particular growth phase in both trials are significantly different (P < 0.05).

The proanthocyanidin content of the different extracts in catechin equilvalent is shown in Figure 3. In the first trial, the aqueous extracts had the lowest proanthocyanidin content which ranged from 3.18 ± 0.62 μg CE/mg in the preflowering stage to 14.09 ± 0.62 μg CE/mg in the flowering stage. The acetone extracts had the highest contents which ranged from 100.91 ± 1.29 μg CE/mg in the preflowering stage to 170 ± 0 in the flowering stages. The methanol extracts however, ranged from 36.82 ± 1.93 μg CE/mg in the preflowering to 100.91 ± 4.50 μg CE/mg in the flowering stage. The same trend was observed in the second trial with the flowering stage having the highest proanthocyanidin content. With regards to the extractive ability of the solvents, the acetone extract of the flowering stage gave the highest yield of polyphenol content for both seasons.

### 3.4 Antioxidant activities

#### 3.4.1 DPPH

**Figure 4:**
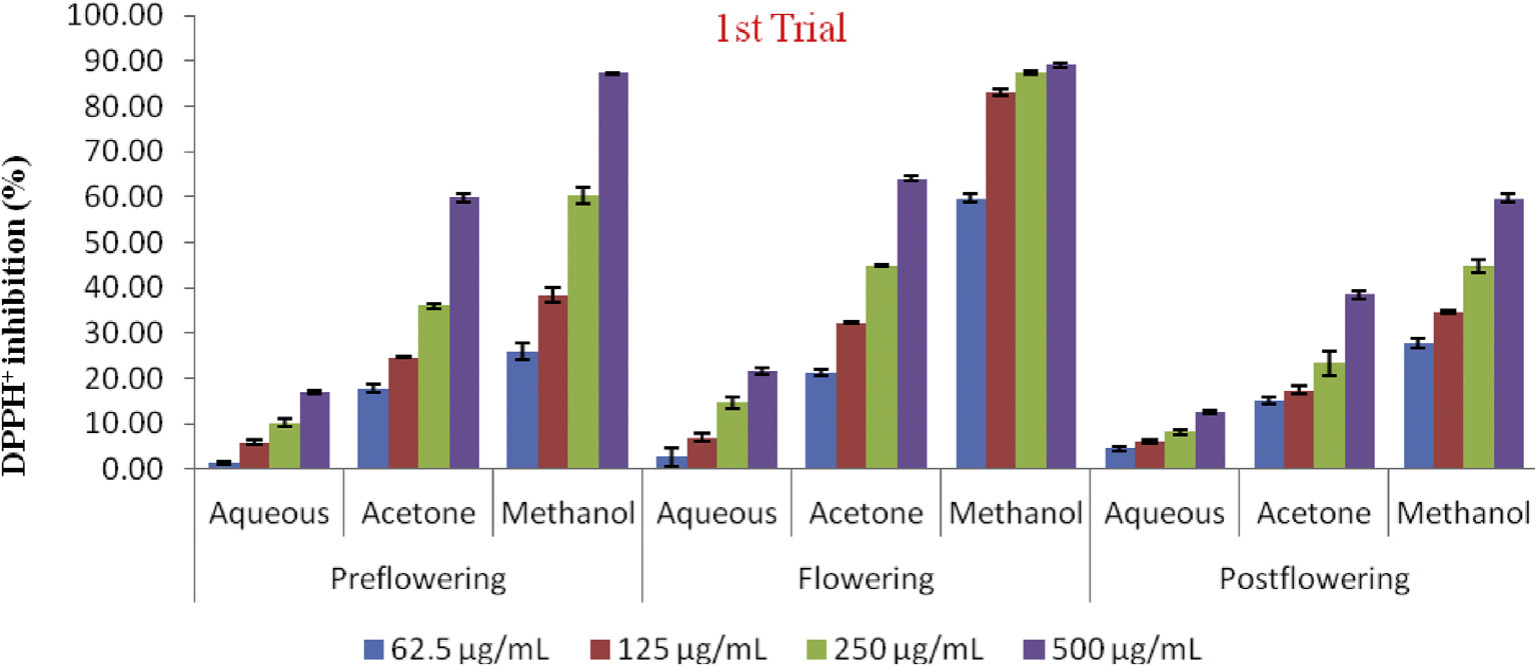
DPPH radical scavenging activity of *C. argentea* extracts at different growth stages of the first trial. Values are means ± SD, n = 3.

**Figure 5:**
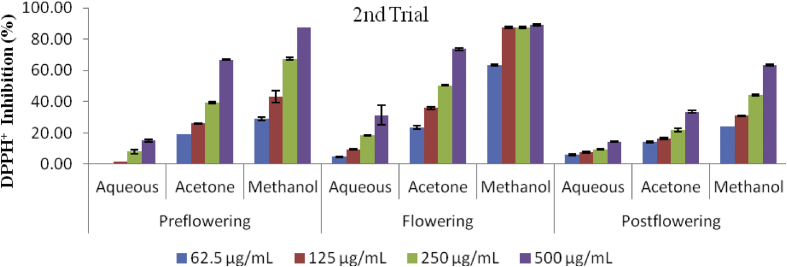
DPPH radical scavenging activity of *C. argentea* extracts at different growth stages of the first trial. Values are means ± SD, n = 3.

**Figure 6:**
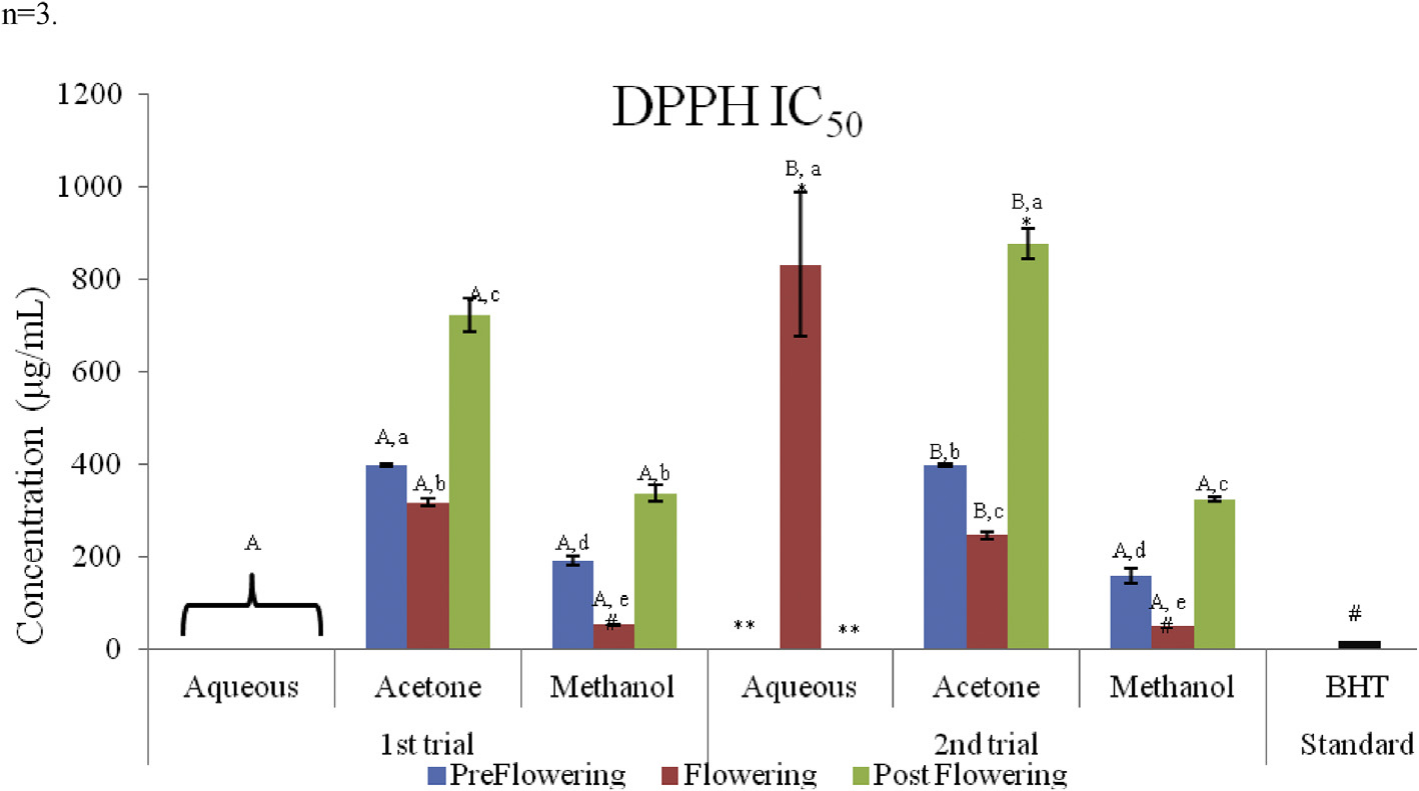
IC_50_ (DPPH) of *C. argentea* extracts at different growth stages of two trials. Values are means ± SD, n = 3; P < 0.05. Different small letters within trials show significant differences (p < 0.05): Different capital letters (A&B) which compare bars of a particular solvent in a particular growth phase in both trials are significantly different. ∗∗IC_50_ values greater than 1000 μg/mL; ∗IC_50_ values greater than 500 μg/mL (highest concentration evaluated); # IC_50_ values less than the lowest concentration (62.5 μg/mL) evaluated.

The antioxidant capacity of different extracts on DPPH radicals and the concentration that inhibited 50% (IC_50_) of the DPPH radicals are presented in Figures 4, 5, and 6. All the extracts scavenged DPPH radicals in a concentration dependent manner. The lowest scavenging activity was observed in the aqueous extracts of all stages of growth in both trials. The methanol extracts exhibited the highest activity ranging from 25.81 ± 1.18 to 87.33 ± 0.14% in the preflowering stage; and from 59.75 ± 0.83 to 89.05 ± 0.48% in the flowering stage; while the methanol post flowering extract (MPST) however, ranged from 27.74 ± 0.96 to 59.75 ± 0.83%. The same trends were also observed for the second trial. The aqueous extracts in all stages of growth in the first trial and in the pre and post flowering stages (APRE and APST) of the second trial were unable to scavenge 50% of the DPPH radicals with IC_50_ values greater than 1000 μg/mL at the concentration tested. Low scavenging activities were also recorded in the post flowering stages of the acetone extract in both trials (722.55 ± 37.373 μg/mL and 878.39 ± 32.81 μg/mL for the 1^st^ and 2^nd^ trial respectively) and also in the flowering stage of the aqueous extract (832.55 ± 99.52 μg/mL) in the second trial which showed IC_50_ values greater than the highest concentration (>500 μg/mL) evaluated but <1000 μg/mL. The scavenging activities of the extracts and BHT are in the order: BHT > MFLW > MPRE > AcFLW > MPST > AcPRE > AcPST > APRE = AFLW = APST in the first trial. The sequence in the second trial was; BHT > MFLW > MPRE > AcPRE > AcFLW > MPST > AcPRE > AcPST > AFLW > APRE = APST (Figure 6).

### 3.5 ABTS activity

**Figure 7:**
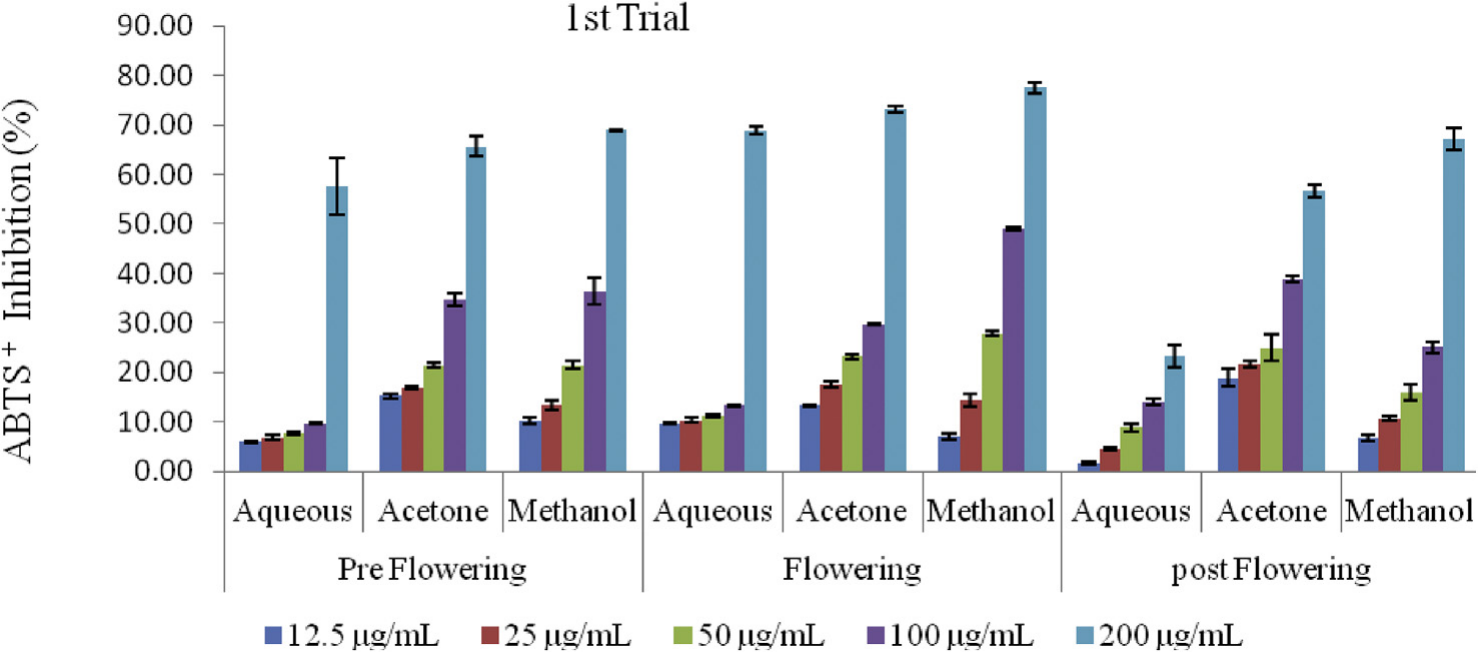
ABTS radical scavenging activity of *C. argentea* at different growth stages of the first trial. Values are means ± SD, n = 3.

**Figure 8:**
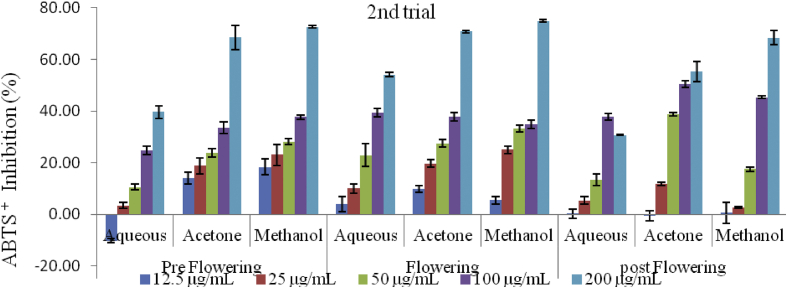
ABTS radical scavenging activity of *C. argentea* at different growth stages of the second trial Values are means ± SD, n = 3.

**Figure 9:**
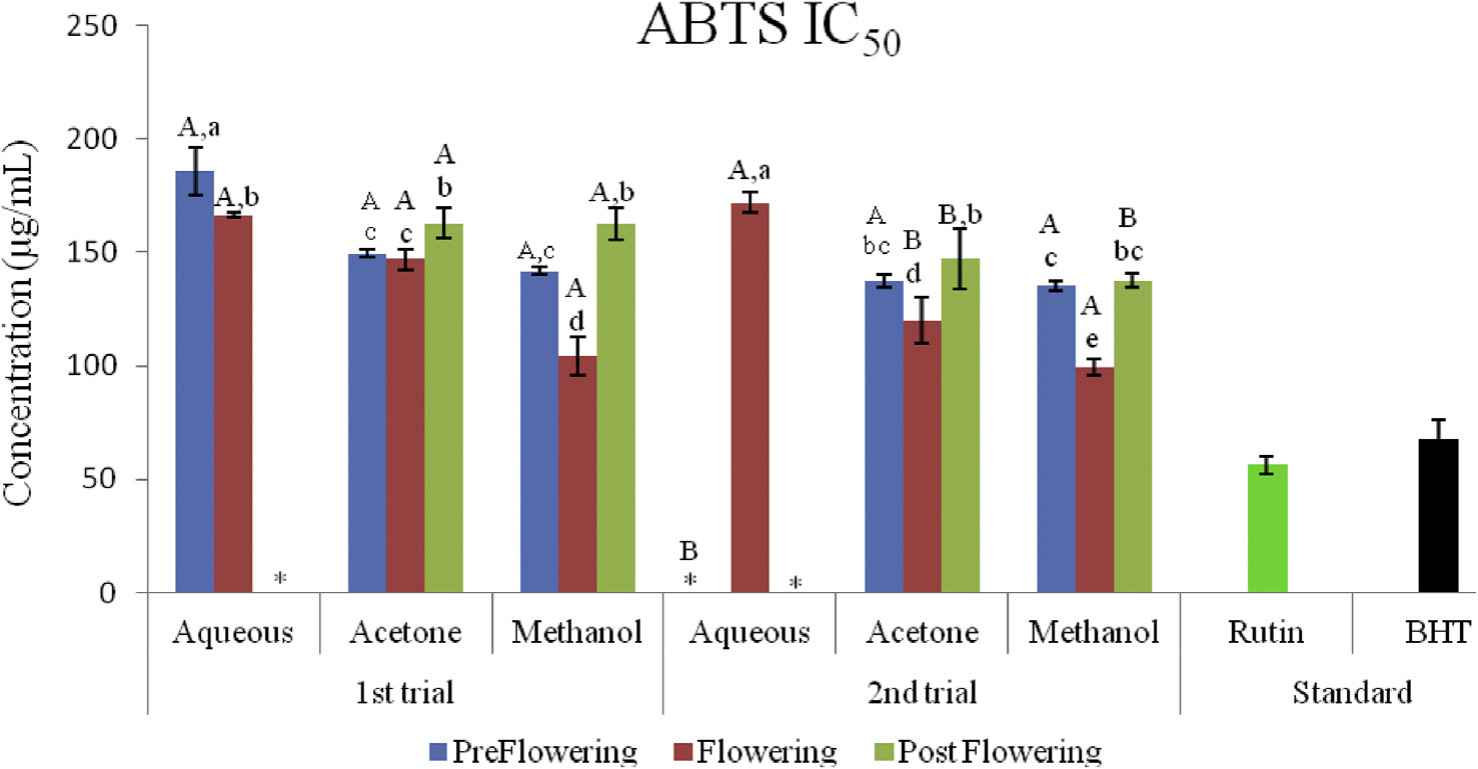
ABTS^+^ IC_50_ of two trials of *C. argentea*. Values are means ± SD, n = 3. Different small letters within trials show significant differences (P < 0.05): Different capital letters (A&B) which compare bars of a particular solvent in a particular growth phase in both trials are significantly different. ∗ values greater than 1000 μg/mL.

The scavenging activities of the extracts on ABTS radical increased with increase in concentration in all stages of growth and in both trials (Figure 7, 8 and 9). The lowest inhibitory activities of 23.33 ± 2.24% and 30.74 ± 0.24% where recorded in the aqueous extracts of the postflowering stage (APST) in the first and second trials respectively. The highest inhibition for the preflowering stage was 57.60 ± 5.65 (in the aqueous extract) followed by 68.95 ± 0.16% (in the methanol extracts) for the first trial; and from 39.55 ± 2.48 (in the aqueous) to 72.63 ± 0.52% (in the methanol extract) for the second trial. Likewise, for the flowering stage, the highest scavenging activities observed in the first and second trials were 77.57 ± 1.06% and 74.84 ± 0.53% respectively. The scavenging activities of the acetone and methanol extracts of the post flowering stages of growth were 56.65 ± 1.19 and 67.14 ± 2.09% for the first trial and 55.32 ± 3.88 and 68.37 ± 2.70% for the second trial respectively.

The ability of the different extracts to scavenge 50% of the ABTS^+^ in the first trial ranged from 104.10 ± 8.59 μg/mL in the methanol extract of the flowering stage to greater than 1000 μg/mL in the aqueous extract of the post flowering while it ranged from 120.02 ± 13.40 μg/mL in the acetone extracts of the flowering stage to greater than 1000 μg/mL in the aqueous extracts of the flowering and post flowering stages in the second trial (Figure 9). The order of decreasing activities based on the IC_50_ is in the order: Rutin > BHT > MFLW > MPRE > AcFLW > AcPRE > MPST > AcPST > AFLW > APRE > APST, in the first trial. The sequence in the second trial was; Rutin > BHT > MFLW > AcFLW > MPRE > AcPRE > MPST > AcPST > AFLW > APRE = APST.

### 3.6 Ferric reducing power (FRAP)

**Figure 10:**
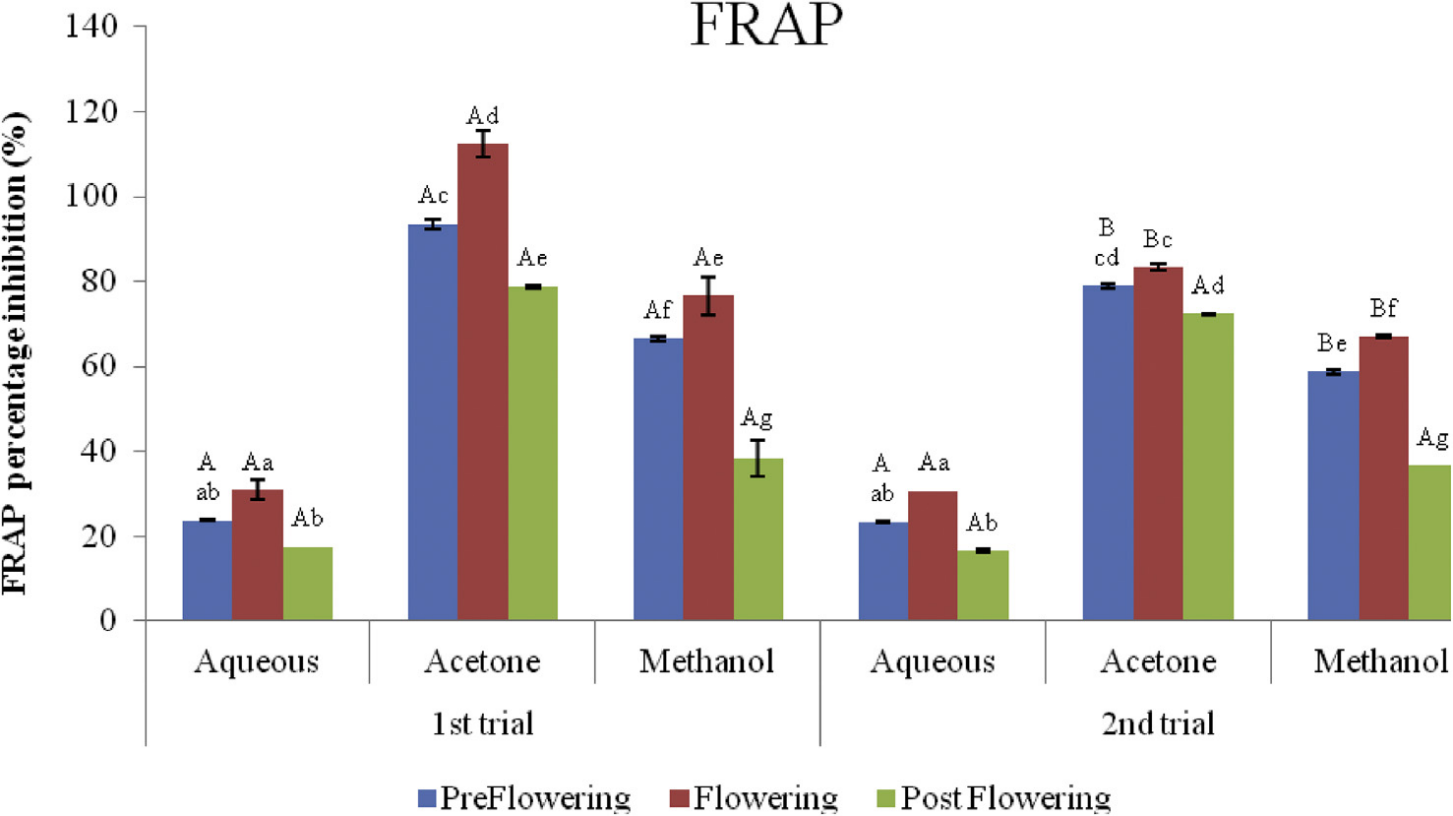
Ferric reducing antioxidant power of two trials of *C. argentea* at different growth stages. Values are means ± SD, n = 3. Different small letters within trials show significant differences (p < 0.05): Different capital letters (A&B) which compare bars of a particular solvent in a particular growth phase in both trials are significantly different.

**Figure 11:**
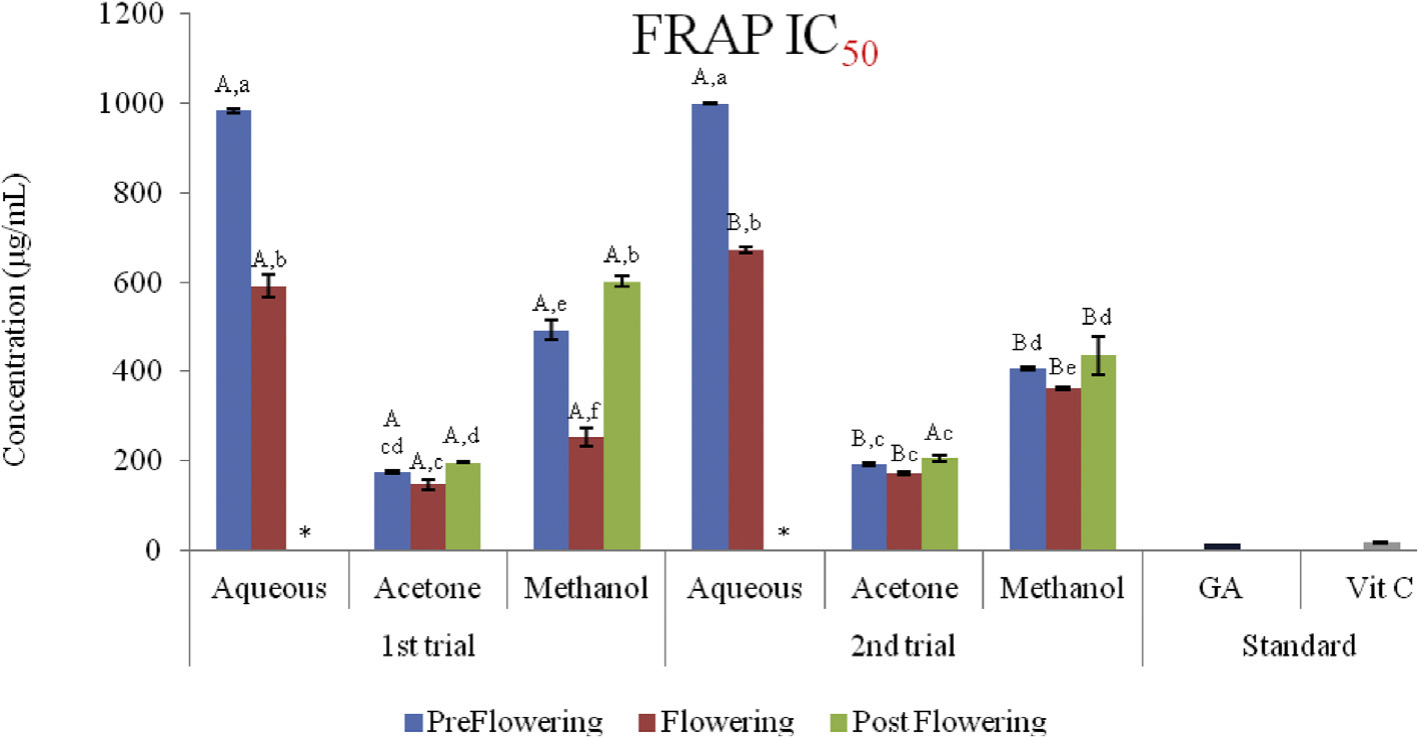
IC_50_ (FRAP) values for *C. argentea* extracts at different growth stages of two trials. Values are means ± SD, n = 3. Different small letters within trials show significant differences (P < 0.05): Different capital letters (A&B) which compare bars of a particular solvent in a particular growth phase in both trials are significantly different. ∗ Values greater than 1000 μg/mL.

There was an increase in the ferric reduction potential of all the extracts with an increase in concentration. The ferric reducing antioxidant power of the extracts ranged from 17.37 ± 0.12 to 112.46 ± 3.24 μg AAE/mg in the first trial and from 16.53 ± 0.36 to 83.31 ± 0.60 μg AAE/mg in the second trial (Figures 10 and 11). FRAP activity of the acetone extracts were, however, significantly higher (P < 0.05) in the first trial (78.73 ± 0.36–112.46 ± 3.24 μg AAE/mg) and second (72. 29 ± 0.12–83.31 ± 0.60 μg AAE/mg) trials than the methanol in the first (36.14 ± 4.31–76.55 ± 4.43 μg AAE/mg) and second (36.70 ± 0.12–66.86 ± 0.36 μg AAE/mg) trials; and the aqueous extracts (17.37 ± 0.12–30.85 ± 2.40 μg AAE/mg and 16.53 ± 0.36–30.68 ± 0.12 μg AAE/mg in the first and second trials respectively).

In comparing the activities within growth phases, the flowering stage in all the solvent extracts in both trials had higher FRAP activity than the preflowering and post flowering stages but were not statistically different (P < 0.05) from the flowering stages in the aqueous extracts of both trials and acetone extracts in the second trial. The postflowering stages however, had the least activity in both trials. The IC_50_ of ferric to ferrous was also determined (Figure 10). The least activities were recorded in the aqueous postflowering extracts (APST) of both trials and preflowering (APRE) of the second trial with IC_50_ greater than 1000 μg/mL. However, the acetone extracts exhibited the best scavenging potential with IC_50_ ranging from 145.82 ± 12.06 μg/mL to 196.561 ± 2.754 μg/mL for the first trial and from 171.73 ± 3.442 μg/mL to 204.351 ± 6.88 μg/mL in the second trial in AcFLW and AcPST respectively. The ferric reducing potential followed the order AcFLW > AcPRE > AcPST > MFLW > MPRE > AFLW > MPST > APRE > APST in the first trials and in this order; AcFLW > AcPRE > APST > MFLW > MPRE > MPST > AFLW > APRE > APST in the second trial.

### 3.7 Phophomolybdenum assay (TAC)

**Figure 12:**
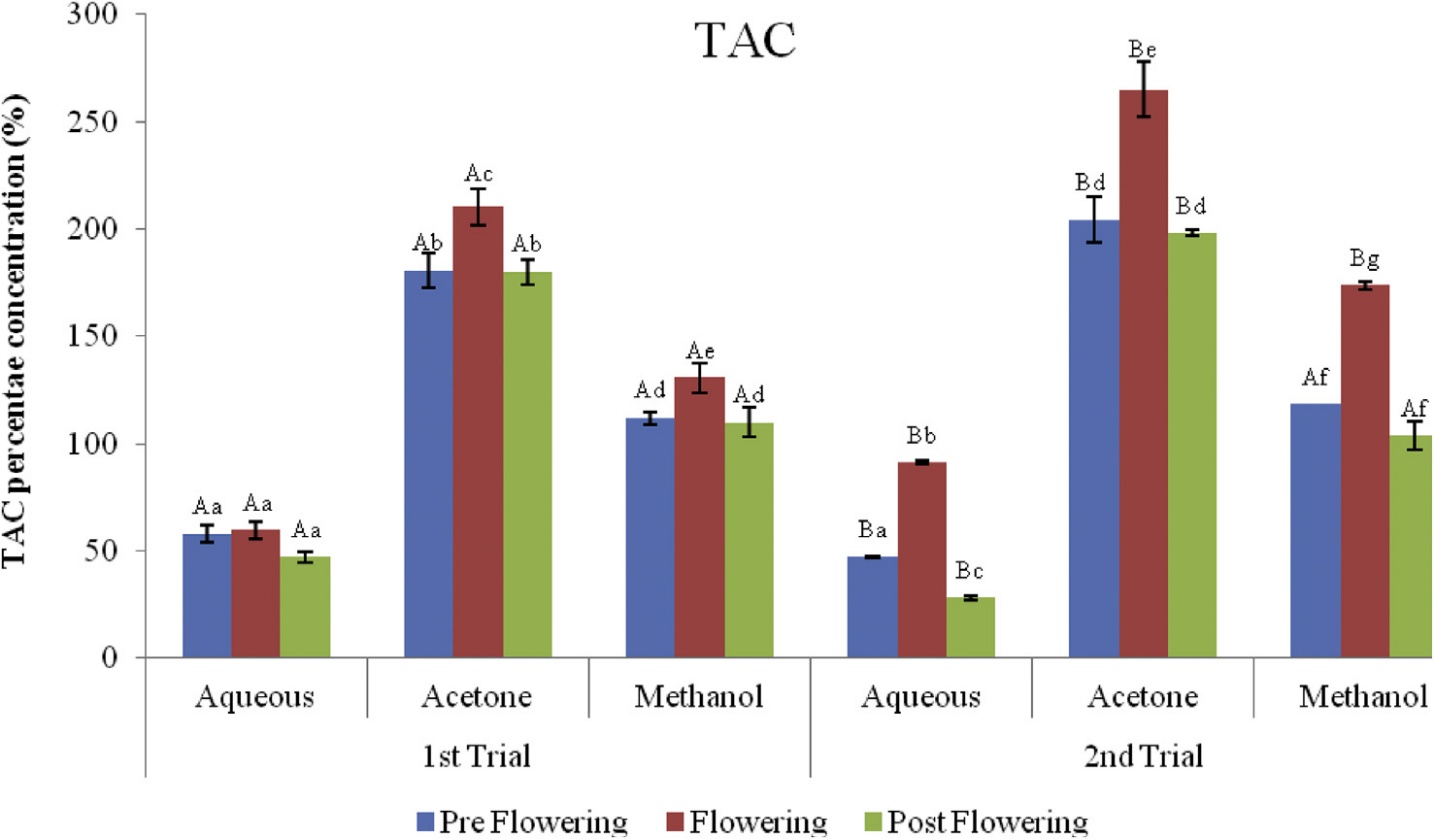
Phosphomolybdenum activity (TAC) of *C. argentea* extracts at different growth stages of two trials. Values are means ± SD, n = 3. Different small letters within trials show significant differences (P < 0.05): Different capital letters (A&B) which compare bars of a particular solvent in a particular growth phase in both trials are significantly different.

**Figure 13:**
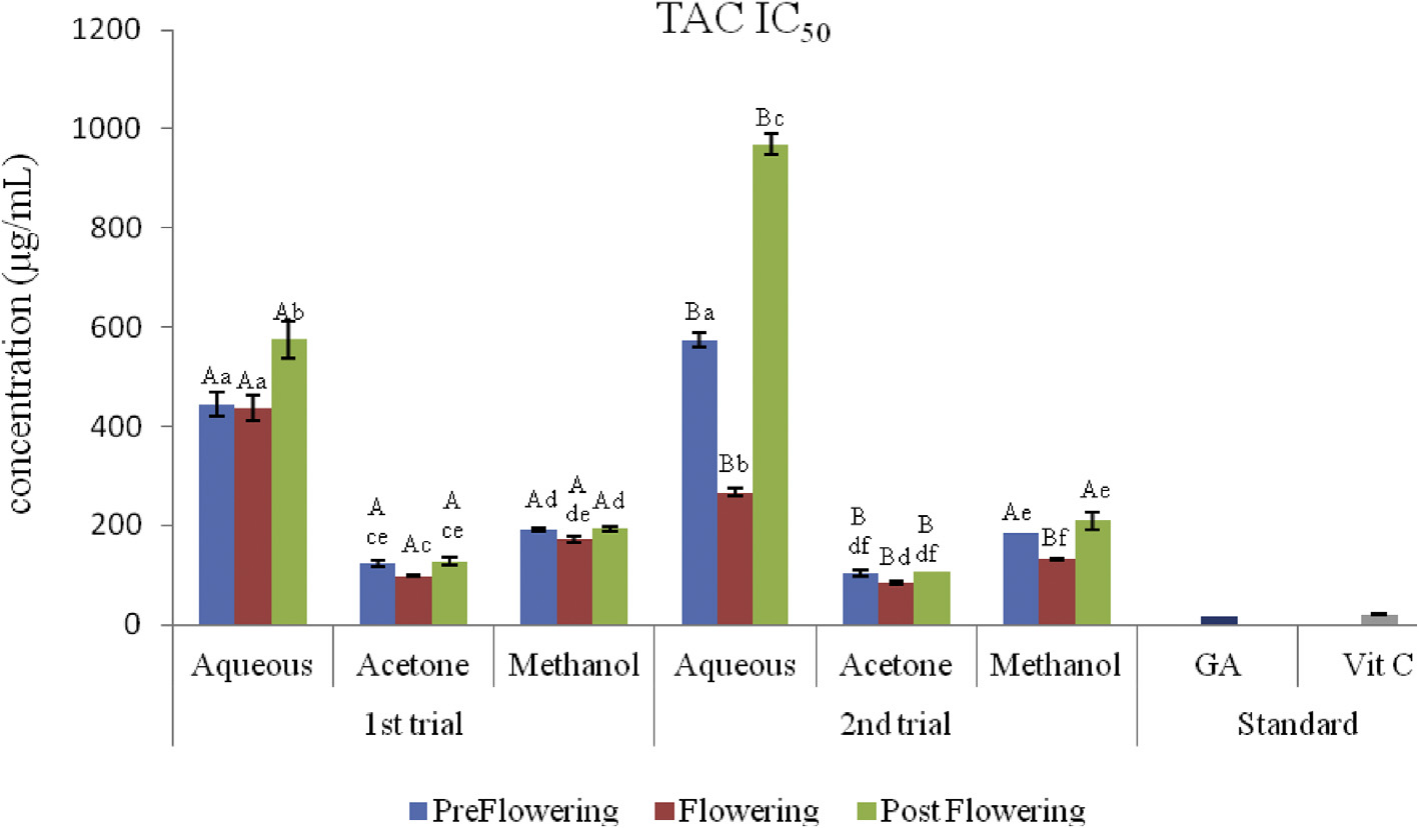
IC_50 (_Phosphomolybdenum activity, TAC) of *C. argentea* at different growth stages of two trials. Values are means ± SD, n = 3. Different small letters within trials show significant differences (P < 0.05): Different capital letters (A&B) which compare bars of a particular solvent in a particular growth phase in both trials are significantly different.

The results of the total antioxidant capacity of the extracts measured by the reduction of molybdenum (VI) to molybdenum(V) is depicted in Figures 12 and 13. The activity of the extract was measured in μg ascorbic acid equivalent per mg (μg AAE/mg). There was an increase in activity with increase in concentration as observed with other assays. The least reduction activity in molybdenum was recorded with the postflowering aqueous extract (47.07 ± 2.64 μg AAE/mg), while the acetone extract of the flowering stage (AcFLW) had the best activity (210.3 ± 8.69 μg AAE/mg) in the first trial. The same trend was observed in the second trial. Postflowering aqueous extract had the least activity (28.00 ± 1.31 μg AAE/mg) while the flowering acetone had the highest activity (265.00 ± 12.82 μg AAE/mg). The ability of the extracts to reduce 50% (IC_50_) of molybdenum (VI) to (V) was also calculated as shown in Figure 12. The activity of the standard drugs and extracts in the first and second trials are; GA > Vit C > AcFLW > AcPRE > AcPST > MFLW > MPRE > MPST > AFLW > APRE > APST and AcFLW > AcPRE > AcPST > MFLW > MPRE > MPST > AFLW > APRE > APST respectively.

## 4 Discussion

Plants synthesize and gather various low and high molecular weight secondary metabolites which have been proven to possess antioxidant capacity (Quideau et al., 2011; Kasote et al., 2015). These naturally occurring biologically active compounds belong to different chemical groups such as the phenols, flavonoids, anthocyanins, diterpenes, isoflavones (Saxena et al., 2013) etc.

Phenolic components (Phenolic acids and flavonoids) have been reported to have the capacity of acting as a reducing agent, singlet oxygen quenchers and hydrogen donors. Due to their redox properties, the antioxidative capacity of plants are chiefly attributed to the presence of these phytocompounds in them (Iswarya Velu et al., 2012). The concentration of these compounds vary according to the plant parts, planting seasons, extracting solvents and growth phases (Lagnika et al., 2016). The series of complex reactions involved in the growth processes of plants ultimately leads to changes in their phytochemistry; for example, the phenolic content of plants may have a rise or steady decrease at the end of maturation (Mahmood et al., 2012). It is therefore crucial to evaluate the phytochemical constituents at different stages of growth so as to avoid any alteration in the medicinal potency of plants. In this study, the results obtained from the phytochemical screening provided evidence that growth phases had impact on the polyphenolic content of *C. argentea.*

The highest phenolic content was observed in the flowering stage irrespective of the season (AcFLW first and second trial). Similar to our findings, a decrease in the phenolic content of *C. argentea* as the plant advanced in age has been reported by Oloyede et al. (2013). Rugna et al. (2008) also obtained similar results for *Smilax campestris* leaves at flowering stage of the plant. The high phenolic content observed in the methanol extract of the preflowering stage (second trial) and all the flowering stages of other solvent of extraction, could be as a result of nutrient depletion in the soil as the plant approaches maturation. There is a trend towards increased phenolics whenever nutrient resources are low in comparison with fixed carbon availability (Jonathan et al., 2016).

Seasonal variations in photoperiod, light intensity and temperature can significantly alter the levels of phenolic compounds in plants. Brasileiro et al. (2015) reported higher levels of phenols for *T*. *triangularae* during the winter period with variations in the phenolic content at different harvest times; similar to our observation in this study. The highest concentration of phenol observed in the acetone extracts of all the plant samples confirms that acetone is highly effective for extracting higher amount of phenolics from plants compared to water and methanol because of its affinity for both lipo- and hydro-philic compounds (Anokwuru et al., 2011). This could also be as a result of the similarity in the polarity of acetone and phenols in the plant (Mahmood et al., 2012).

Flavonoids are an important class of plant pigments, naturally found in fruits and vegetables. They are known to possess a series of biological properties due to their ability to interfere in the formation of free radicals. Flavonoids present in the diet have been found to reduce blood cholesterol levels; thus allieviating the risk of coronary diseases (Kumar and Pandey, 2013). The flavonoid content in this study was highest in the flowering stage in all the solvents and in both trials. In agreement with our findings, several researchers reported an increase in the concentration of flavonoid content in different plants such as *Nigella sativa* (Zribi et al., 2014), *Aquilaria beccariana* (Anwar et al., 2017), and *Ziziphora clinopodioides* (Ding et al., 2014) during their flowering stage. However, an inverse trend for flavonoid content was reported for *Cynara cardunculus* (Zeipina et al., 2016). The high concentration of flavonoids at the flowering stage could be attributed to the fact that flavonoids play major role in fruit colouration, ultra-violet protection, pigmentation and aroma in flower production (Saxena et al., 2013). As the plant prepares for flowering, more flavonoids are likely to be synthesized. Biosynthesis of secondary metabolites like flavonoids during the flowering period of plants could also be a defense mechanism against pests that may attack the flowers (Petrussa et al., 2013) and a means of attracting potential pollinators.

The proanthocyanidin content of the plant sample was highest in the acetone extract of the flowering stage and lowest in the aqueous extract of the flowering stage second trial. The increase in the proanthocyanidin as the plant approaches maturation with a steady decrease during senescence is in line with the observation of Denev and Yordanov (2013) and that of Del Bubba et al. (2009) for *Diospyros kaki*. The same reason as stated for the flavonoids could be responsible for the similar trends observed with the proanthocyanidin because they belong to the same class of compounds.

Overall, the highest phytochemical content was recorded in the second trial. This could be the effect of change in temperature in the environment, since the concentrations of various secondary metabolites are strongly dependent on the growing conditions and this invariably influences the metabolic pathways of plant (Akula and Ravishankar, 2011). Considering the fact that *C. argentea* is a tropical plant (Kanu et al., 2017), low temperature has been implicated as the most harmful abiotic factor for plants growing in the temperate regions. During winter, plant metabolism is redirected towards the synthesis of cryoprotectant molecules which induce increase in the production of phytochemicals (Janska et al., 2010).

Antioxidants inhibit free radicals using different reaction mechanisms, which includes singlet oxygen quenching in the presence of co-factors (metals chelators), hydrogen donation, reduction of hydrogen perioxide, interception of lipid peroxyl radical etc. Due to the differences in the mode of action of antioxidants, the antioxidant potential of *C. argentea* crude extracts was evaluated using DPPH, ABTS, FRAP and TAC assays. The high DPPH radical scavenging potential recorded for the methanol extract of the flowering stages in the 1^st^ and 2^nd^ trials may be due to the high levels of phenolic content recorded at this growth stage. Antioxidants that possess hydrogen atom donating capacity have the ability to quench DPPH radicals by converting them to colourless products with a corresponding decrease in absorbance (Illavarasan et al., 2005). Polyphenolic with hydroxyl groups are good hydrogen donors; they donate H^+^ to neutralise the activity of radicals (Mathew et al., 2015). This could also account for the lowest value of IC_50_ (49.4 ± 0.29 μg/mL methanol 2^nd^ trial) recorded at this growth stage; which implies that the methanol extract at the flowering stage of growth had a good mopping action on DPPH radical.

Similar to the DPPH, the methanol extracts of both 1^st^ and 2^nd^ trials of the flowering stage had a better scavenging potential against ABTS^+^ with IC_50_ values of (104.10 ± 8.59 and 98.98 ± 3.39 μg/mL). Although the acetone extract had the highest phenol, flavonoids and proanthocyanidin contents, the methanol extract exhibited more scavenging activity on both DPPH and ABTS radicals. This could be as a result of methanol being able to extract more of the phytocompounds in the plant responsible for the mopping of these radicals and the synergistic relationships between these compounds to favor greater antioxidant activity (Ahmed et al., 2015).

The FRAP model involves the reduction of the Fe^3+^/ferricyanide complex to greenish ferrous form after reacting with hydrogen atom to break the free radical chain. The acetone extract in both trials showed more inhibitory activity on the ferric radicals. As observed in previous assays in this study, the flowering stages of the solvent exhibited better reducing power. There was a positive correlation of the phenol, flavonoids and proanthocyanidin content with FRAP. Several authors have reported that phenols and flavonoids are excellent hydrogen donating antioxidants which have protective effect on oxidative damages induced by the hydroxyl radicals (Saeed et al., 2012; Nimse and Pal, 2015; Zhang et al., 2016). This implies that *C. argentea* extract possess both hydrogen donating and oxygen quenching capacities.

The total antioxidant activity (TAC) is based on the formation of green phosphate/molybdenum(V) complex at acid pH and the subsequent reduction of molybdenum (VI) to molybdenum(V) by the plant extract. The effect of the different solvents showed that the acetone extract of the flowering stage acetone extract of the 2^nd^ trial had greater potency with an IC_50_ value of 85.6 μg AAE/mg_._ Similar to the FRAP results, there was a positive correlation of the phenol and flavonoid content of the acetone extracts in the flowering stage with the IC_50_ values of TAC.

## 5 Conclusion

To obtain an increased level of phyto-compounds from *C. argentea* L. for medicinal purposes, the age of the plant at harvest and the time of season are of great importance as it provides limitless prospects for drug discovery. This study confirms that quantitative differences exist in the concentration of phytochemical compounds and antioxidant capacities at different seasons and phases of growth. The optimum period to harvest these vegetable for biological activities is the flowering stage, when the concentrations of polyphenolic compounds are highest. The antioxidant activities of *C. argentea* reported in this study shows that the vegetable has the potential to prevent and/or manage conditions caused by oxidative stress. Furthermore, *C. argentea* at early flowering stage could serve as a good source of phytonutrients which could play a major role in promoting health and nutrition.

## Declarations

### Author contribution statement

Gloria Aderonke Otunola: Conceived and designed the experiments; Analyzed and interpreted the data; Contributed reagents, materials, analysis tools or data.

Oluwafunmilayo Dorcas Adegbaju: Conceived and designed the experiments; Performed the experiments; Analyzed and interpreted the data; Wrote the paper.

Anthony Jide Afolayan: Conceived and designed the experiments; Contributed reagents, materials, analysis tools or data.

### Funding statement

This research did not receive any specific grant from funding agencies in the public, commercial, or not-for-profit sectors.

### Competing interest statement

The authors declare no conflict of interest.

### Additional information

No additional information is available for this paper.

## References

[bib2] Agbor G.A., Vinson J.A., Oben J.E., Ngogang J.Y. (2010). Antioxidant effect of herbs and spices on copper mediated oxidation of lower and very low density lipoprotein. Chin. J. Nat. Med..

[bib3] Ahmed D., Khan M.M., Saeed R. (2015). Comparative analysis of phenolics, flavonoids, and antioxidant and antibacterial potential of methanolic, hexanic and aqueous extracts from Adiantum caudatum leaves. Antioxidants.

[bib4] Akula R., Ravishankar G.A. (2011). Influence of abiotic stress signals on secondary metabolites in plants. Plant Signal. Behav..

[bib5] Anokwuru C.P., Anyasor G.N., Ajibaye O., Fakoya O., Okebugwu P. (2011). Effect of extraction solvents on phenolic, flavonoid and antioxidant activities of three Nigerian medicinal plants. Nature and Science. J. Evid. Based Compl. Altern. Med..

[bib6] Anwar K., Rahmanto B., Triyasmono L., Rizki M.I., Halwany W., Lestari F. (2017). The influence of leaf age on total phenolic, flavonoids, and free radical scavenging capacity of Aquilaria beccariana. Res. J. Pharmaceut. Biol. Chem. Sci..

[bib7] Brasileiro B.G., Leite J.P.V., Casali V.W.D., Pizziolo V.R., Coelho O.G.L. (2015). The influence of planting and harvesting times on the total phenolic content and antioxidant activity of Talinum triangulare (Jacq.). Acta Sci. Agron..

[bib8] Del Bubba M., Giordani E., Pippucci L., Cincinelli A., Checchini L., Galvan P. (2009). Changes in tannins, ascorbic acid and sugar content in astringent persimmons during on-tree growth and ripening and in response to different postharvest treatments. J. Food Compos. Anal..

[bib9] Denev P., Yordanov A. (2013). Total polyphenol, proanthocyanidin and flavonoid content, carbohydrate composition and antioxidant activity of persimmon (Diospyros kaki L.) fruit in relation to cultivar and maturity stage. Bulg. J. Agric. Sci..

[bib10] Ding W., Yang T., Liu F., Tian S. (2014). Effect of different growth stages of Ziziphora clinopodioides Lam. on its chemical composition. Pharmacogn. Mag..

[bib11] Gupta A., Sheikh S., Yadav N. (2013). Development of underutilised *celosia* argentea based value added product and its impact on haemoglobin status of adolescent girls. Acta Hortic..

[bib12] Illavarasan R., Mallika M., Venkataraman S. (2005). Anti-inflammatory and antioxidant activities of Cassia fistula Linn bark extracts. Afr. J. Tradit., Compl. Altern. Med..

[bib13] Iswarya Velu A.R., Gopalakrishnan D., Manivannan B., Sathiavelu M., Arunachalam S. (2012). Comparison of antioxidant activity and total phenolic content of Amaranthus tristis and *Celosia argentea* var *spicata*. Asian Pac. J. Trop Biomed..

[bib14] Janská A., Maršík P., Zelenková S., Ovesná J. (2010). Cold stress and acclimation–what is important for metabolic adjustment?. J. Plant Biol..

[bib15] Jayanthi P., Lalitha P. (2011). Reducing power of the solvent extracts of *Eichhornia crassipes* (Mart.) Solms. Int. J. Pharm. Pharmaceut. Sci..

[bib16] Jonathan R., Sundqvist M.K., Gundale M.J., Giesler R., Wardle D.A. (2016). Effects of elevation and nitrogen and phosphorus fertilization on plant defense compounds in subarctic tundra heath vegetation. Funct. Ecol..

[bib17] Kanu C.L., Owoeye O., Imosemi I.O., Malomo A.O. (2017). A review of the multifaceted usefulness of *celosia argentea* Linn. Euro. J. Pharm. Med. Res..

[bib18] Kasote D.M., Katyare S.S., Hegde M.V., Bae H. (2015). Significance of antioxidant potential of plants and its relevance to therapeutic applications. Int. J. Biol. Sci..

[bib19] Khan R.A., Khan M.R., Sahreen S., Ahmed M. (2012). Evaluation of phenoliccontents and antioxidant activity of various solvent extracts of Sonchus asper (L) Hill. Chem. Cent. J..

[bib20] Kumar S., Pandey A.K. (2013). Chemistry and biological activities of flavonoids: an overview. Sci. World J..

[bib21] Kurutas E.B. (2015). The importance of antioxidants which play the role in cellular response against oxidative/nitrosative stress: current state. Nutr. J..

[bib22] Lagnika L., Amoussa A.M.O., Adjileye R.A., Laleye A., Sanni A. (2016). Antimicrobial, antioxidant, toxicity and phytochemical assessment of extracts from A cmella uliginosa, a leafy-vegetable consumed in Bénin, West Africa. BMC Compl. Altern. Med..

[bib23] Lamien-Meda A., Nell M., Lohwasser U., Börner A., Franz C., Novak J. (2010). Investigation of antioxidant and rosmarinic acid variation in the sage collection of the genebank in Gatersleben. J. Agric. Food Chem..

[bib24] Mahmood T., Anwar F., Abbas M., Saari N. (2012). Effect of maturity on phenolics (phenolic acids and flavonoids) profile of strawberry cultivars and mulberry species from Pakistan. Int. J. Mol. Sci..

[bib25] Mathew S., Abraham T.E., Zakaria Z.A. (2015). Reactivity of phenolic compounds towards free radicals under in vitro conditions. J. Food Sci. Technol..

[bib26] Nidavani R.B., Mahalakshmi A.M., Seema M., Krishna K.L. (2014). Pharmacology of *Celosia argentea* L. J. Atoms Mol..

[bib27] Nimse S.B., Pal D. (2015). Free radicals, natural antioxidants, and their reaction mechanisms. RSC Adv..

[bib28] Ohikhena F.U., Wintola O.A., Afolayan A.J. (2018). Quantitative phytochemical constituents and antioxidant activities of the mistletoe, phragmanthera capitata (sprengel) balle extracted with different solvents. Pharmacogn. Res..

[bib29] Oloyede F.M., Oloyede F.A., Obuotore E.M. (2013). Effect of plant maturity on the antioxidant profile of Amaranthus cruentus L. And celosia argentea L. Bull. Env. Pharmacol. Life Sci..

[bib30] Olugbami J.O., Gbadegesin M.A., Odunola O.A. (2015). *In vitro* free radical scavenging and antioxidant properties of ethanol extract of Terminalia glaucescens. Pharmacogn. Res..

[bib31] Petrussa E., Braidot E., Zancani M., Peresson C., Bertolini A., Patui S., Vianello A. (2013). Plant flavonoids—biosynthesis, transport and involvement in stress responses. Int. J. Mol. Sci..

[bib32] Quideau S., Deffieux D., Douat-Casassus C., Pouysegu L. (2011). Plant polyphenols: chemical properties, biological activities, and synthesis. Angew. Chem. Int. Ed..

[bib33] Rugna A.Z., Ricco R., Gurni A., Wagner M. (2008). Variation in leaves polyphenol content in Smilax campestris Griseb. - smilacaceae - according to their development. Lat. Am. J. Pharm..

[bib34] Saeed N., Khan M.R., Shabbir M. (2012). Antioxidant activity, total phenolic and total flavonoid contents of whole plant extracts Torilis leptophylla L. BMC Compl. Alternative Med..

[bib35] Saxena M., Saxena J., Nema R., Singh D., Gupta A. (2013). Phytochemistry of medicinal plants. J. Pharmacogn. Phytochem..

[bib36] Tang Y., Xin H.L., Guo M.L. (2016). Review on research of the phytochemistry and pharmacological activities of *Celosia argentea*. Rev. Bras. Farmacogn..

[bib37] Tsao R., Khanizadeh S., Dale A. (2006). Designer fruits and vegetables with enriched phytochemicals for human health. Can. J. Plant Sci..

[bib38] Unuofin J.O., Otunola G.A., Afolayan A.J. (2018). Polyphenolic content, antioxidant and antimicrobial activities of vernonia mespilifolia less. Used in folk medicine in the eastern Cape province. Evid. Based Compl. Altern. Med..

[bib39] Varadharaj V., Muniyappan J. (2017). Phytochemical and phytotherapeutic properties of celosia species-A review. Int. J. Pharmacogn. Phytochem. Res..

[bib41] Zeipina S., Lepse L., Alsina I. (2016). The effect of agroecological factors on yield and flavonoids content of globe artichoke. Res. Rural Dev..

[bib40] Zhang H., Li X., Wu K., Wang M., Liu P., Wang X., Deng R. (2016). Antioxidant activities and chemical constituents of flavonoids from the flower of Paeonia ostii. Molecules.

[bib42] Zribi I., Omezzine F., Haouala R. (2014). Variation in phytochemical constituents and allelopathic potential of **Nigella sativa** with developmental stages. S. Afri. J. Bot..

